# Crystal structure of (*E*)-*N*-(3,4-di­meth­oxy­benzyl­idene)morpholin-4-amine

**DOI:** 10.1107/S160053681401678X

**Published:** 2014-08-01

**Authors:** Sevim Türktekin Çelikesir, Mehmet Akkurt, Aliasghar Jarrahpour, Orhan Büyükgüngör

**Affiliations:** a Department of Physics, Faculty of Sciences, Erciyes University, 38039 Kayseri, Turkey; b Department of Chemistry, College of Sciences, Shiraz University, 71454 Shiraz, Iran; c Department of Physics, Faculty of Arts and Sciences, Ondokuz Mayıs University, 55139 Samsun, Turkey

**Keywords:** crystal structure, hydrogen bonding, C—H⋯π inter­actions, Schiff bases, morpholin-4-amine

## Abstract

In the title compound, C_13_H_18_N_2_O_3_, the benzene ring makes a dihedral angle of 17.19 (11)° with the least-squares plane formed by the four C atoms of the morpholine ring, which adopts a chair conformation. In the crystal, C—H⋯N hydrogen bonds link the mol­ecules into supramolecular chains running along a 2_1_ screw axis parallel to the *b-*axis direction. Weak C—H⋯π inter­actions are also observed.

## Related literature   

For the structures of related compounds, see: Akkurt *et al.* (2013 ▶, 2014 ▶). For ring-puckering parameters, see: Cremer & Pople (1975 ▶).
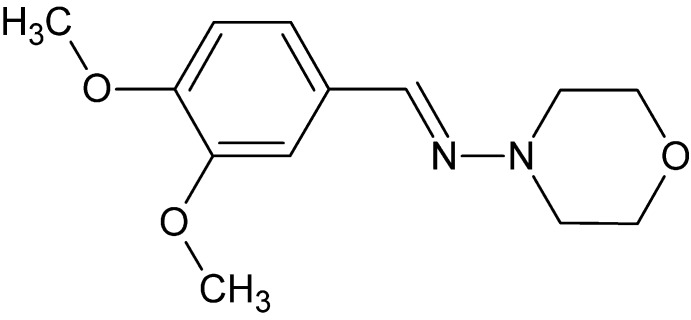


## Experimental   

### Crystal data  

C_13_H_18_N_2_O_3_
*M* *_r_* = 250.29
Monoclinic, 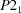

*a* = 9.1644 (6) Å
*b* = 6.0277 (6) Å
*c* = 13.1327 (9) Å
β = 109.989 (5)°
*V* = 681.75 (10) Å^3^
*Z* = 2
Mo *K*α radiation
μ = 0.09 mm^−1^
*T* = 296 K
0.58 × 0.42 × 0.24 mm

### Data collection  

Stoe IPDS 2 diffractometer
Absorption correction: integration (*X-RED32*; Stoe & Cie, 2002 ▶) *T*_min_ = 0.962, *T*_max_ = 0.983
8493 measured reflections
3219 independent reflections
2071 reflections with *I* > 2σ(*I*)
*R*_int_ = 0.229

### Refinement  

*R*[*F* ^2^ > 2σ(*F* ^2^)] = 0.046
*wR*(*F* ^2^) = 0.106
*S* = 1.00
3219 reflections
164 parameters
1 restraint
H-atom parameters constrained
Δρ_max_ = 0.09 e Å^−3^
Δρ_min_ = −0.18 e Å^−3^
Absolute structure: Flack (1983 ▶), 1353 Friedel pairs
Absolute structure parameter: −0.4 (19)

### 

Data collection: *X-AREA* (Stoe & Cie, 2002 ▶); cell refinement: *X-AREA*; data reduction: *X-RED32* (Stoe & Cie, 2002 ▶); program(s) used to solve structure: *SHELXS97* (Sheldrick, 2008 ▶); program(s) used to refine structure: *SHELXL97* (Sheldrick, 2008 ▶); molecular graphics: *ORTEP-3 for Windows* (Farrugia, 2012 ▶); software used to prepare material for publication: *WinGX* (Farrugia, 2012 ▶), *PARST* (Nardelli, 1983 ▶) and *PLATON* (Spek, 2009 ▶).

## Supplementary Material

Crystal structure: contains datablock(s) global, I. DOI: 10.1107/S160053681401678X/rz5130sup1.cif

Structure factors: contains datablock(s) I. DOI: 10.1107/S160053681401678X/rz5130Isup2.hkl

Supporting information file. DOI: 10.1107/S160053681401678X/rz5130Isup3.cml

**Fig. 1.**
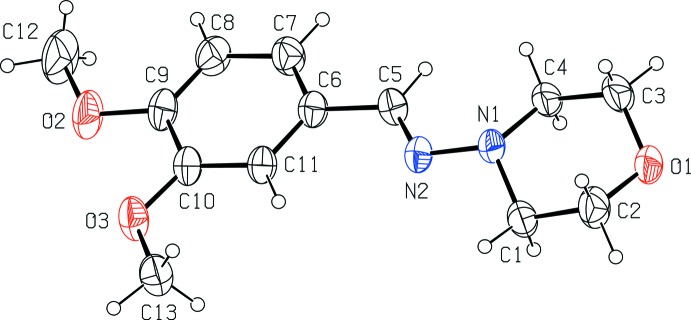
The molecular structure of the title compound with 30% probability displacement ellipsoids.

**Fig. 2.**
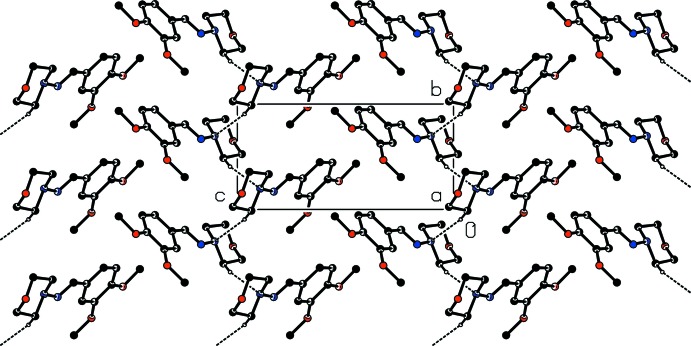
Packing diagram of the title compound viewed down the a axis. Hydrogen bonds are indicated by dashed lines. For clarity, H atoms not participating in hydrogen bonding are omitted.

. DOI: 10.1107/S160053681401678X/rz5130fig1.tif
The mol­ecular structure of the title compound with 30% probability displacement ellipsoids.

a . DOI: 10.1107/S160053681401678X/rz5130fig2.tif
Packing diagram of the title compound viewed down the *a* axis. Hydrogen bonds are indicated by dashed lines. For clarity, H atoms not participating in hydrogen bonding are omitted.

CCDC reference: 1015028

Additional supporting information:  crystallographic information; 3D view; checkCIF report

## Figures and Tables

**Table 1:** Hydrogen-bond geometry (Å, °) *Cg*1 is the centroid of the C6–C11 benzene ring.

*D*—H⋯*A*	*D*—H	H⋯*A*	*D*⋯*A*	*D*—H⋯*A*
C1—H1*B*⋯N1^i^	0.97	2.61	3.542 (3)	161
C8—H8⋯*Cg*1^ii^	0.93	2.87	3.576 (3)	134

Symmetry codes: (i) 
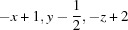
; (ii) 
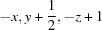
.

## supplementary crystallographic information

### S1. Comment

As part of our continuing interest in the design and chemistry of Schiff bases containing a morpholine moiety, the title compound has been synthesized and its crystal structure is reported herein. In the title compound (Fig. 1), the benzene ring (C6–C11) makes a dihedral angle of 17.19 (11)° with the least-squares plane formed by the four C atoms of the morpholine ring (C1–C4/N1/O1), which adopts a chair conformation [the puckering parameters (Cremer & Pople, 1975) are *Q*_T_ = 0.557 (3) Å, θ = 177.2 (3)°, φ = 177 (7)°]. The N1–N2–C5–C6, C10–C9–O2–C12 and C9–C10–O3–C13 torsion angles are -173.7 (2), 178.2 (3) and -178.9 (3)°, respectively. The bond lengths and bond angles are normal and comparable with those reported for related compounds (Akkurt *et al.* 2013, 2014). In the crystal structure, molecules are linked by intermolecular C—H···N hydrogen bonds forming supramolecular chains running along a 2_1_ screw axis parallel to the [010] direction (Table 1, Fig. 2). In addition, weak C—H. . .π interactions also occur (Table 1).

### S2. Experimental

Reaction of 3,4-dimethoxybenzaldehyde (1.0 mmol) with morpholin-4-amine (1.0 mmol) in refluxing ethanol gave the title compound. Recrystallization from ethanol gave colourless crystals in 85 % yield. M.p.: 345-347 K. IR (KBr) cm^-1^:1604 (C=N). ^1^H-NMR (250 MHz, CDCl_3_), δ (ppm): 3.06 (CH_2_-N, t, 4H, J=5 Hz), 3.79 (CH_2_-O, t, 4H, J=5 Hz), 3.83 (2OMe, s, 6H), 6.73 (aromatic H, d, 1H, J=7.5 Hz), 6.93 (aromatic H, d, 1H, J=7.5 Hz), 7.49 (aromatic H, s, 1H), 7.81 (HC=N, s, 1H). ^13^CNMR (62.9 MHz, CDCl_3_), δ (p.p.m): 52.1 (CH_2_-N), 55.8 (2OMe), 66.4 (CH_2_-O), 107.4-137.0 (aromatic carbons), 149.6 (C═N).

### S3. Refinement

All H atoms were located geometrically with C—H = 0.93-0.97 Å, and refined using a riding model with *U*_iso_(H) = 1.2 *U*_eq_(C) or 1.5 *U*_eq_(C) for methyl H atoms. Owing to the poor quality of the crystal, the data obtained were rather poor and the value of *R*_int_ remained high (0.229).

### Crystal data

**Table:**

C_13_H_18_N_2_O_3_	*F*(000) = 268
*M**_r_* = 250.29	*D*_x_ = 1.219 Mg m^−^^3^
Monoclinic, *P*2_1_	Mo *K*α radiation, λ = 0.71073 Å
Hall symbol: P 2yb	Cell parameters from 8791 reflections
*a* = 9.1644 (6) Å	θ = 3.3–28.7°
*b* = 6.0277 (6) Å	µ = 0.09 mm^−^^1^
*c* = 13.1327 (9) Å	*T* = 296 K
β = 109.989 (5)°	Block, colourless
*V* = 681.75 (10) Å^3^	0.58 × 0.42 × 0.24 mm
*Z* = 2	

### Data collection

**Table:**

Stoe IPDS 2 diffractometer	3219 independent reflections
Radiation source: sealed X-ray tube, 12 x 0.4 mm long-fine focus	2071 reflections with *I* > 2σ(*I*)
Plane graphite monochromator	*R*_int_ = 0.229
Detector resolution: 6.67 pixels mm^-1^	θ_max_ = 28.4°, θ_min_ = 3.3°
ω scans	*h* = −12→12
Absorption correction: integration (*X-RED32*; Stoe & Cie, 2002)	*k* = −8→7
*T*_min_ = 0.962, *T*_max_ = 0.983	*l* = −17→17
8493 measured reflections	

### Refinement

**Table:**

Refinement on *F*^2^	Hydrogen site location: inferred from neighbouring sites
Least-squares matrix: full	H-atom parameters constrained
*R*[*F*^2^ > 2σ(*F*^2^)] = 0.046	*w* = 1/[σ^2^(*F*_o_^2^) + (0.0511*P*)^2^] where *P* = (*F*_o_^2^ + 2*F*_c_^2^)/3
*wR*(*F*^2^) = 0.106	(Δ/σ)_max_ < 0.001
*S* = 1.00	Δρ_max_ = 0.09 e Å^−^^3^
3219 reflections	Δρ_min_ = −0.18 e Å^−^^3^
164 parameters	Absolute structure: Flack (1983), 1353 Friedel pairs
1 restraint	Absolute structure parameter: −0.4 (19)

### Special details

**Table:**

Geometry. Bond distances, angles *etc*. have been calculated using the rounded fractional coordinates. All su's are estimated from the variances of the (full) variance-covariance matrix. The cell e.s.d.'s are taken into account in the estimation of distances, angles and torsion angles
Refinement. Refinement on *F*^2^ for ALL reflections except those flagged by the user for potential systematic errors. Weighted *R*-factors *wR* and all goodnesses of fit *S* are based on *F*^2^, conventional *R*-factors *R* are based on *F*, with *F* set to zero for negative *F*^2^. The observed criterion of *F*^2^ > σ(*F*^2^) is used only for calculating -*R*-factor-obs *etc*. and is not relevant to the choice of reflections for refinement. *R*-factors based on *F*^2^ are statistically about twice as large as those based on *F*, and *R*-factors based on ALL data will be even larger.

### Fractional atomic coordinates and isotropic or equivalent isotropic displacement parameters (Å^2^)

**Table:**

	*x*	*y*	*z*	*U*_iso_*/*U*_eq_	
O1	0.87001 (19)	0.1514 (4)	0.97941 (17)	0.0790 (8)	
O2	−0.3171 (2)	0.2682 (4)	0.53060 (17)	0.0904 (9)	
O3	−0.19462 (19)	−0.0304 (4)	0.67524 (15)	0.0760 (7)	
N1	0.5456 (2)	0.1976 (4)	0.89414 (16)	0.0551 (7)	
N2	0.3907 (2)	0.1691 (4)	0.83192 (16)	0.0559 (7)	
C1	0.6164 (3)	−0.0123 (5)	0.9354 (2)	0.0690 (10)	
C2	0.7736 (3)	0.0250 (7)	1.0207 (3)	0.0825 (13)	
C3	0.8006 (3)	0.3569 (6)	0.9444 (3)	0.0961 (13)	
C4	0.6442 (3)	0.3349 (5)	0.8551 (3)	0.0741 (10)	
C5	0.3240 (3)	0.3067 (5)	0.7579 (2)	0.0618 (9)	
C6	0.1559 (3)	0.2948 (5)	0.6976 (2)	0.0577 (9)	
C7	0.0901 (3)	0.4480 (6)	0.6185 (2)	0.0717 (10)	
C8	−0.0684 (3)	0.4447 (6)	0.5604 (2)	0.0749 (10)	
C9	−0.1606 (3)	0.2862 (6)	0.5817 (2)	0.0677 (9)	
C10	−0.0941 (3)	0.1245 (5)	0.66164 (19)	0.0581 (9)	
C11	0.0628 (3)	0.1315 (5)	0.71871 (18)	0.0564 (9)	
C12	−0.3889 (4)	0.4346 (9)	0.4511 (4)	0.1281 (18)	
C13	−0.1323 (3)	−0.1930 (6)	0.7562 (3)	0.0776 (10)	
H1A	0.62780	−0.09990	0.87660	0.0830*	
H1B	0.55040	−0.09360	0.96640	0.0830*	
H2A	0.76090	0.10140	1.08210	0.0990*	
H2B	0.82230	−0.11720	1.04580	0.0990*	
H3A	0.86890	0.44530	0.91820	0.1150*	
H3B	0.78720	0.43450	1.00540	0.1150*	
H4A	0.59760	0.48020	0.83530	0.0890*	
H4B	0.65700	0.26800	0.79150	0.0890*	
H5	0.38240	0.41810	0.74130	0.0740*	
H7	0.15210	0.55610	0.60340	0.0860*	
H8	−0.11160	0.55040	0.50700	0.0900*	
H11	0.10700	0.02590	0.77200	0.0680*	
H12A	−0.49830	0.40600	0.42040	0.1920*	
H12B	−0.34400	0.43100	0.39490	0.1920*	
H12C	−0.37230	0.57810	0.48490	0.1920*	
H13A	−0.21360	−0.29130	0.75850	0.1160*	
H13B	−0.08730	−0.12190	0.82530	0.1160*	
H13C	−0.05390	−0.27630	0.73970	0.1160*	

### Atomic displacement parameters (Å^2^)

**Table:**

	*U*^11^	*U*^22^	*U*^33^	*U*^12^	*U*^13^	*U*^23^
O1	0.0392 (9)	0.0779 (15)	0.1082 (16)	0.0027 (11)	0.0100 (9)	0.0184 (13)
O2	0.0436 (10)	0.127 (2)	0.0832 (13)	0.0098 (12)	−0.0007 (9)	0.0218 (14)
O3	0.0453 (9)	0.1042 (16)	0.0703 (11)	−0.0062 (12)	0.0093 (8)	0.0151 (13)
N1	0.0362 (9)	0.0605 (14)	0.0612 (11)	−0.0022 (10)	0.0070 (9)	−0.0039 (11)
N2	0.0393 (9)	0.0702 (15)	0.0539 (11)	0.0014 (11)	0.0105 (8)	−0.0019 (12)
C1	0.0479 (13)	0.070 (2)	0.0838 (18)	−0.0075 (15)	0.0156 (12)	0.0121 (18)
C2	0.0496 (14)	0.093 (3)	0.091 (2)	−0.0002 (17)	0.0060 (14)	0.023 (2)
C3	0.0467 (15)	0.074 (2)	0.138 (3)	−0.0134 (15)	−0.0066 (17)	0.020 (2)
C4	0.0451 (14)	0.0609 (18)	0.102 (2)	−0.0048 (14)	0.0068 (14)	0.0169 (18)
C5	0.0459 (12)	0.0722 (19)	0.0632 (15)	−0.0077 (14)	0.0133 (12)	−0.0020 (16)
C6	0.0446 (12)	0.071 (2)	0.0521 (13)	0.0005 (14)	0.0094 (10)	−0.0019 (14)
C7	0.0562 (14)	0.079 (2)	0.0709 (16)	−0.0042 (16)	0.0100 (13)	0.0092 (17)
C8	0.0605 (15)	0.084 (2)	0.0666 (16)	0.0101 (18)	0.0042 (13)	0.0193 (17)
C9	0.0428 (13)	0.094 (2)	0.0570 (15)	0.0066 (14)	0.0050 (11)	−0.0003 (16)
C10	0.0416 (11)	0.081 (2)	0.0488 (12)	−0.0002 (14)	0.0119 (10)	−0.0001 (15)
C11	0.0436 (11)	0.075 (2)	0.0456 (12)	0.0016 (14)	0.0087 (10)	0.0017 (14)
C12	0.0602 (18)	0.159 (4)	0.130 (3)	0.022 (3)	−0.013 (2)	0.050 (3)
C13	0.0599 (16)	0.092 (2)	0.0796 (18)	−0.0077 (17)	0.0223 (14)	0.0102 (18)

### Geometric parameters (Å, º)

**Table:**

O1—C2	1.408 (4)	C1—H1A	0.9700
O1—C3	1.397 (4)	C1—H1B	0.9700
O2—C9	1.364 (3)	C2—H2A	0.9700
O2—C12	1.435 (6)	C2—H2B	0.9700
O3—C10	1.366 (4)	C3—H3A	0.9700
O3—C13	1.415 (4)	C3—H3B	0.9700
N1—N2	1.385 (3)	C4—H4A	0.9700
N1—C1	1.441 (4)	C4—H4B	0.9700
N1—C4	1.443 (4)	C5—H5	0.9300
N2—C5	1.265 (3)	C7—H7	0.9300
C1—C2	1.509 (4)	C8—H8	0.9300
C3—C4	1.516 (5)	C11—H11	0.9300
C5—C6	1.474 (4)	C12—H12A	0.9600
C6—C7	1.367 (4)	C12—H12B	0.9600
C6—C11	1.391 (4)	C12—H12C	0.9600
C7—C8	1.391 (4)	C13—H13A	0.9600
C8—C9	1.366 (5)	C13—H13B	0.9600
C9—C10	1.409 (4)	C13—H13C	0.9600
C10—C11	1.376 (4)		
			
C2—O1—C3	109.4 (2)	C1—C2—H2B	109.00
C9—O2—C12	116.5 (3)	H2A—C2—H2B	108.00
C10—O3—C13	117.1 (2)	O1—C3—H3A	109.00
N2—N1—C1	110.6 (2)	O1—C3—H3B	109.00
N2—N1—C4	120.1 (2)	C4—C3—H3A	109.00
C1—N1—C4	112.4 (2)	C4—C3—H3B	109.00
N1—N2—C5	120.0 (2)	H3A—C3—H3B	108.00
N1—C1—C2	110.0 (3)	N1—C4—H4A	110.00
O1—C2—C1	111.1 (3)	N1—C4—H4B	110.00
O1—C3—C4	112.4 (3)	C3—C4—H4A	110.00
N1—C4—C3	108.5 (3)	C3—C4—H4B	110.00
N2—C5—C6	121.5 (3)	H4A—C4—H4B	108.00
C5—C6—C7	119.0 (3)	N2—C5—H5	119.00
C5—C6—C11	121.8 (2)	C6—C5—H5	119.00
C7—C6—C11	119.2 (3)	C6—C7—H7	120.00
C6—C7—C8	120.9 (3)	C8—C7—H7	120.00
C7—C8—C9	120.0 (3)	C7—C8—H8	120.00
O2—C9—C8	125.1 (3)	C9—C8—H8	120.00
O2—C9—C10	115.2 (3)	C6—C11—H11	120.00
C8—C9—C10	119.7 (3)	C10—C11—H11	120.00
O3—C10—C9	115.5 (2)	O2—C12—H12A	109.00
O3—C10—C11	125.1 (2)	O2—C12—H12B	109.00
C9—C10—C11	119.3 (3)	O2—C12—H12C	109.00
C6—C11—C10	120.7 (3)	H12A—C12—H12B	109.00
N1—C1—H1A	110.00	H12A—C12—H12C	109.00
N1—C1—H1B	110.00	H12B—C12—H12C	110.00
C2—C1—H1A	110.00	O3—C13—H13A	109.00
C2—C1—H1B	110.00	O3—C13—H13B	109.00
H1A—C1—H1B	108.00	O3—C13—H13C	109.00
O1—C2—H2A	109.00	H13A—C13—H13B	109.00
O1—C2—H2B	109.00	H13A—C13—H13C	109.00
C1—C2—H2A	109.00	H13B—C13—H13C	110.00
			
C2—O1—C3—C4	−60.7 (3)	N2—C5—C6—C7	180.0 (3)
C3—O1—C2—C1	59.7 (4)	N2—C5—C6—C11	−0.3 (4)
C12—O2—C9—C8	−2.2 (5)	C5—C6—C11—C10	179.7 (3)
C12—O2—C9—C10	178.2 (3)	C11—C6—C7—C8	1.0 (4)
C13—O3—C10—C9	−178.9 (3)	C7—C6—C11—C10	−0.6 (4)
C13—O3—C10—C11	1.5 (4)	C5—C6—C7—C8	−179.2 (3)
N2—N1—C1—C2	−168.4 (2)	C6—C7—C8—C9	−0.3 (5)
C4—N1—N2—C5	−20.1 (4)	C7—C8—C9—C10	−0.9 (4)
N2—N1—C4—C3	173.6 (2)	C7—C8—C9—O2	179.4 (3)
C4—N1—C1—C2	54.3 (3)	O2—C9—C10—O3	1.4 (4)
C1—N1—N2—C5	−153.6 (2)	C8—C9—C10—C11	1.3 (4)
C1—N1—C4—C3	−53.6 (3)	O2—C9—C10—C11	−179.0 (2)
N1—N2—C5—C6	−173.7 (2)	C8—C9—C10—O3	−178.3 (3)
N1—C1—C2—O1	−56.7 (3)	O3—C10—C11—C6	179.1 (3)
O1—C3—C4—N1	57.1 (3)	C9—C10—C11—C6	−0.6 (4)

### Hydrogen-bond geometry (Å, º)

*Cg*1 is the centroid of the C6–C11 benzene ring.

**Table:**

*D*—H···*A*	*D*—H	H···*A*	*D*···*A*	*D*—H···*A*
C1—H1*B*···N1^i^	0.97	2.61	3.542 (3)	161
C8—H8···*Cg*1^ii^	0.93	2.87	3.576 (3)	134

Symmetry codes: (i) −*x*+1, *y*−1/2, −*z*+2; (ii) −*x*, *y*+1/2, −*z*+1.
