# Crystal structure of (*E*)-*N*-(3,4-di­meth­oxy­benzyl­idene)morpholin-4-amine. Corrigendum

**DOI:** 10.1107/S2056989014026589

**Published:** 2015-01-01

**Authors:** Sevim Türktekin Çelikesir, Mehmet Akkurt, Aliasghar Jarrahpour, Mehdi Mohammadi Chermahini, Orhan Büyükgüngör

**Affiliations:** a Department of Physics, Faculty of Sciences, Erciyes University, 38039 Kayseri, Turkey; b Department of Chemistry, College of Sciences, Shiraz University, 71454 Shiraz, Iran; c Department of Physics, Faculty of Arts and Sciences, Ondokuz Mayıs University, 55139 Samsun, Turkey

## Abstract

The list of authors in the paper by Çelikesir *et al.* [*Acta Cryst.* (2014), E**70**, o935] is corrected.

## Abstract 2

Corrigendum to *Acta Cryst.* (2014), E**70**, o935.

In the paper by Çelikesir *et al.* (2014) ▸, the list of authors was incorrect. The correct list is given above.
