# Supplementary figures and images for: Crystal structure of (E)-N-(3,4-di­meth­oxy­benzyl­idene)morpholin-4-amine
Source: Acta Crystallogr Sect E Struct Rep Online. 2014 Aug 1;70(Pt 9):o935. doi: 10.1107/S160053681401678X (PMC4186114; doi:10.1107/S160053681401678X)

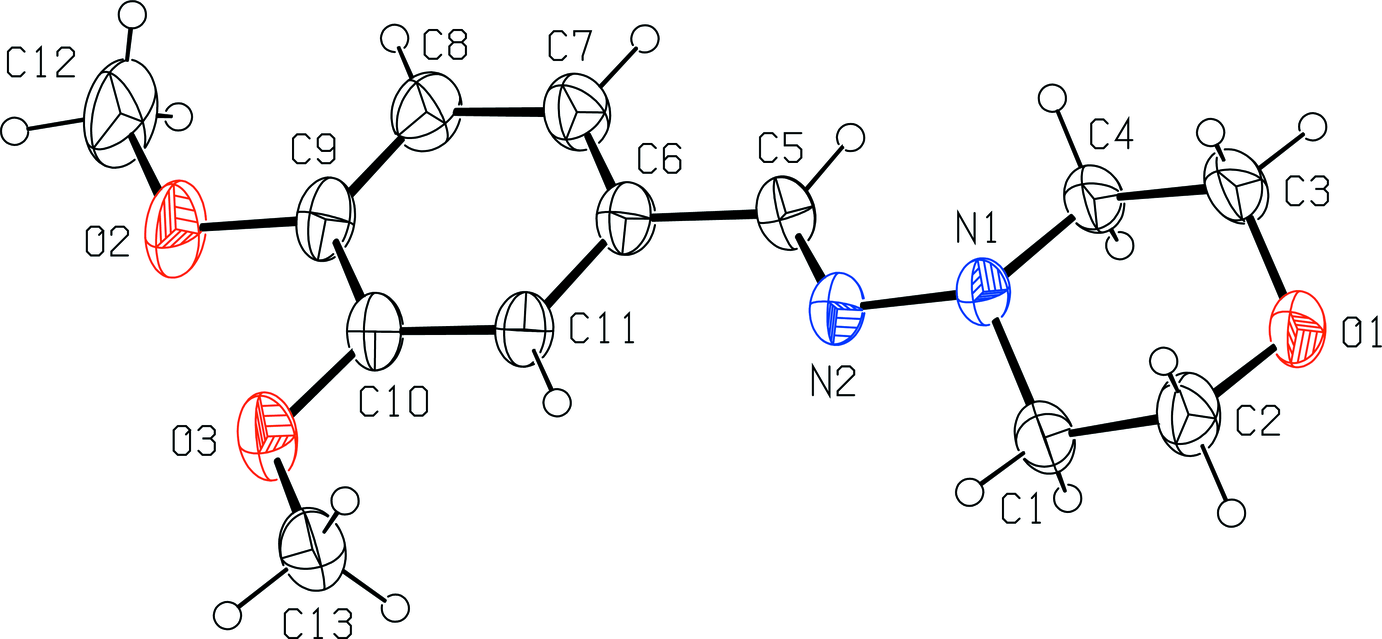

Supplement: Supplementary file 4 [file e-70-0o935-fig1.tif]

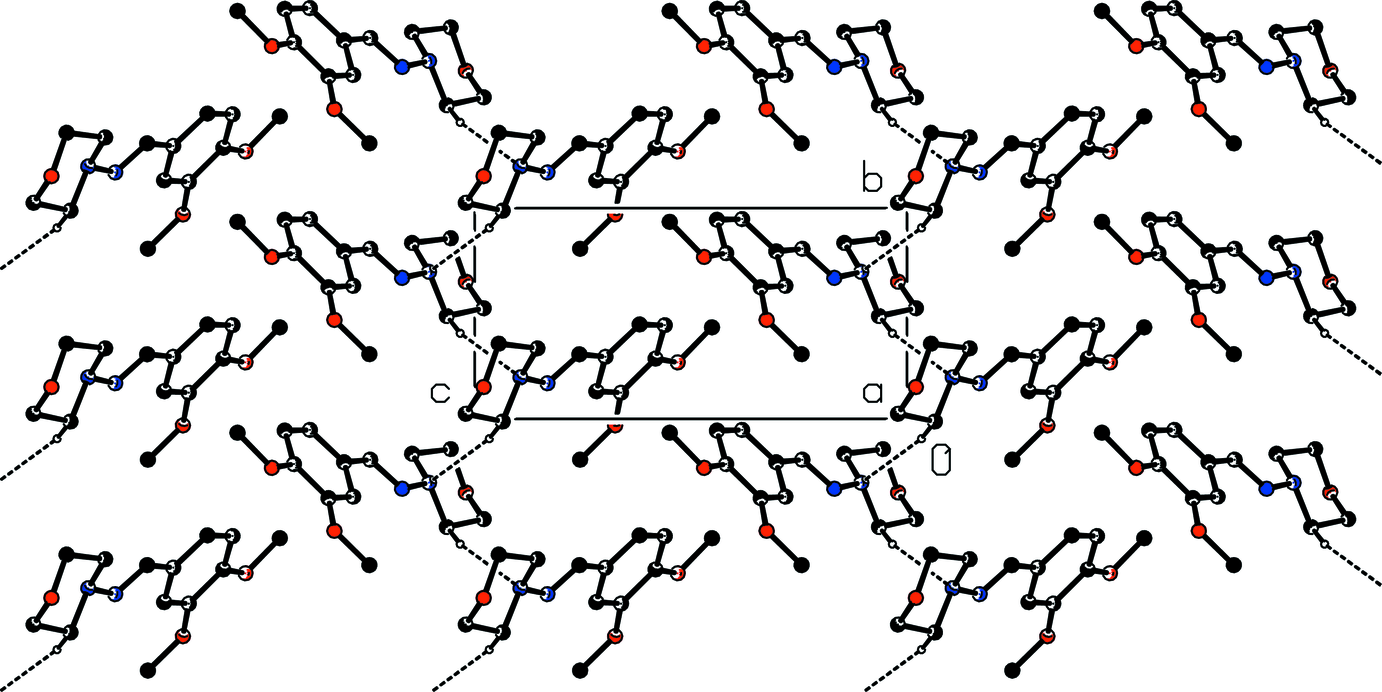

Supplement: Supplementary file 5 [file e-70-0o935-fig2.tif]
